# A microbial detection array (MDA) for viral and bacterial detection

**DOI:** 10.1186/1471-2164-11-668

**Published:** 2010-11-25

**Authors:** Shea N Gardner, Crystal J Jaing, Kevin S McLoughlin, Tom R Slezak

**Affiliations:** 1 Global Security, Lawrence Livermore National Laboratory, Livermore, CA 94551, USA

## Abstract

**Background:**

Identifying the bacteria and viruses present in a complex sample is useful in disease diagnostics, product safety, environmental characterization, and research. Array-based methods have proven utility to detect in a single assay at a reasonable cost any microbe from the thousands that have been sequenced.

**Methods:**

We designed a pan-Microbial Detection Array (MDA) to detect all known viruses (including phages), bacteria and plasmids and developed a novel statistical analysis method to identify mixtures of organisms from complex samples hybridized to the array. The array has broader coverage of bacterial and viral targets and is based on more recent sequence data and more probes per target than other microbial detection/discovery arrays in the literature. Family-specific probes were selected for all sequenced viral and bacterial complete genomes, segments, and plasmids. Probes were designed to tolerate some sequence variation to enable detection of divergent species with homology to sequenced organisms, and to have no significant matches to the human genome sequence.

**Results:**

In blinded testing on spiked samples with single or multiple viruses, the MDA was able to correctly identify species or strains. In clinical fecal, serum, and respiratory samples, the MDA was able to detect and characterize multiple viruses, phage, and bacteria in a sample to the family and species level, as confirmed by PCR.

**Conclusions:**

The MDA can be used to identify the suite of viruses and bacteria present in complex samples.

## Background

For microbial surveillance and discovery based on nucleic acids from an uncharacterized sample, sequencing provides the most in-depth and unbiased information. However, the expense and time required for sequencing using high throughput methods such as 454 (Roche), Solexa (Illumina), or SOLiD (Life Technologies) can make these methods prohibitive for routine use, especially when the resources required for data processing and analysis are taken into account. Viruses at low concentration may be missed if coverage is insufficient, and host sequence may make up 90% or more of the reads [1]. At the other end of the cost spectrum, PCR assays are very fast and sensitive, but have limited capacity for multiplexing (to test for the presence of several organisms simultaneously). They are also intolerant of primer-target mismatches; this is an advantage for detecting a microbe whose sequence is precisely known, but a great disadvantage for discovery of novel species, or for detecting variant strains of a known species.

Microarrays span a middle ground between sequencing and PCR, offering high probe density for detection of diverse and possibly unexpected targets, costs in the hundreds rather than thousands of dollars per sample, and processing times on the order of 24 hours or less. Arrays can be designed with a combination of *detection *and *discovery *probes, which respectively target species-specific regions (for precise characterization of known pathogens), or more conserved regions (to enable detection of novel organisms with some homology to previously sequenced organisms). Compared to sequencing, microarray analysis has the disadvantage that probes designed from known sequences are unlikely to detect truly novel organisms lacking homology to those sequences. However, microbial genomes are difficult and time-consuming to characterize when they have no similarity to known sequences, so the ability of sequencing to identify novel genomes has limited practical benefit in a rapid diagnostic context.

Microbial detection arrays provide an assessment of known pathogens complementary to that provided by a functional gene array, such as the array to detect virulence and antibiotic resistance gene families described in [2]. Detection arrays can tell what organisms are present, while functional arrays can tell what capabilities those organisms might possess. Together these tools may be applied to detect antibiotic resistant or virulent pathogen variants, natural chimeras, or genetically engineered organisms with unusual gene content.

Detection/discovery microarrays have proven useful in identifying or discovering viruses with homology to known species [3-8]. They may thus be used to guide the selection of a subset of samples for further analysis by sequencing. Arrays can also be applied to study clinical samples for which PCR diagnostics have been uninformative. Often, the cause of a clinically severe infection is unknown, complicating the decision of whether to treat with antibiotics, antivirals, or other therapies. With optimization, and sufficiently high pathogen titers, we have successfully generated array results in as little as 2 hours (unpublished data). Moreover, arrays can assist in uncovering co-infections with more than one organism. Microbial detection arrays can also be used to check isolate stocks and vaccines for adventitious contaminants [9]. Finally, arrays can be used to assess the complexity of a metagenomic sample to determine the desired depth for sequencing, potentially saving costs on low complexity samples. Microarrays may reveal greater diversity in complex environmental samples than sequencing of a typical sized clone library [10]. Until the processing time and cost of high throughput sequencing (including data analysis) decreases enough to be feasible for large numbers of samples at sufficient depth, microarrays will continue to be a valuable tool.

Several groups have designed microarrays containing probes for microbial detection, discovery, or a combination of both [3-8,10-20]. Their approaches may be distinguished according to the range of pathogens targeted, the probe design strategy, and the array platform used.

The ViroChip discovery array was one of the first to target a broad range of pathogens; it is best known for its role in characterizing SARS as a coronavirus [4,5,14]. It was designed by selecting probes from regions conserved in the same family or genus based on BLAST nucleotide sequence similarity, so that all complete viral genomes available when it was designed (2002) were represented by 3 probes. Later generations of the ViroChip had 5-10 probes per genome and covered a larger set of genomes; version 3 of the array included approximately 22,000 probes. It was fabricated using spotted oligo technology, which limits the number of probes that can be included on one array.

Chou et al. [11] designed conserved genus probes and species specific probes covering 53 viral families and 214 genera, requiring two probes per virus. They empirically tested a subset of 72 probes targeting the coronavirus, flavivirus, and enterovirus families against pure cultures of six species, although they did not examine clinical samples.

Palacios et al. [12] built the GreeneChipPm, an array targeting vertebrate viruses and rRNA sequences of fungi, bacteria, and protozoa, containing approximately 30,000 probes. It is an oligonucleotide array fabricated using the Agilent ink-jet system. Viral probes were designed to target a minimum of three genomic regions for each family or genus, including at least one highly conserved region coding for polymerase or structural proteins, and two or more variable regions. Bacterial, fungal and protozoan probes were exclusively designed against variable segments of rRNA genes (16S for bacteria, 18S for eukaryotes), flanked by highly conserved regions, so that the target regions could be amplified with a small number of specific PCR primers. Other groups have followed similar strategies for bacterial array design [10,15-17]. The lack of any similar genes universally conserved among viruses precludes using this approach for viral target amplification.

Similar arrays designed by the same group include the GreeneChipVr [3] (targeting viruses only) and the GreeneChipResp [18] (targeting respiratory pathogens). The GreeneChipPm array successfully identified viruses at the species level, and was used to implicate *Plasmodium falciparum *for an unexplained death. It performed less well with bacterial samples, because probes against the 16S rRNA variable regions frequently cross-hybridized across taxa, so that some bacteria could only be identified at family or class resolution.

Array design to fit all probes to span an entire kingdom on a single microarray demands substantial investment in probe selection algorithms. Jabado et al. [19] developed probe design software used to target conserved amino acid regions in viruses using profile hidden Markov models and motif analysis, for which uniqueness relative to non-targets was not a consideration. To our knowledge, experimental data has not yet been published for this array. Satya et al. [20] built a software pipeline TOFI-beta for selecting target-specific probes that are unique relative to a database of non-targets, without requirements for conservation within a set of multiple targets, and illustrated its application in silico using two bacterial genomes.

In this study we describe a comprehensive, high-density oligonucleotide array for detection and discovery of bacteria and viruses. The large number of features on this array, together with an efficient probe design strategy, made it possible to cover all complete bacterial and viral genome sequences with a much larger number of probes per target than previously reported array designs. We discuss the process used for array design, and report the results of testing the array against known mixtures of DNA and RNA viruses, as well as a variety of clinical (fecal, sputum, and serum) samples. We also present a novel statistical algorithm for analysis of detection/discovery arrays, which combines a predictive model of probe hybridization with a greedy likelihood maximization procedure to identify the combination of targets in a complex sample that best explains the observed probe intensity pattern.

## Results

### Array design

**Figure 1:**
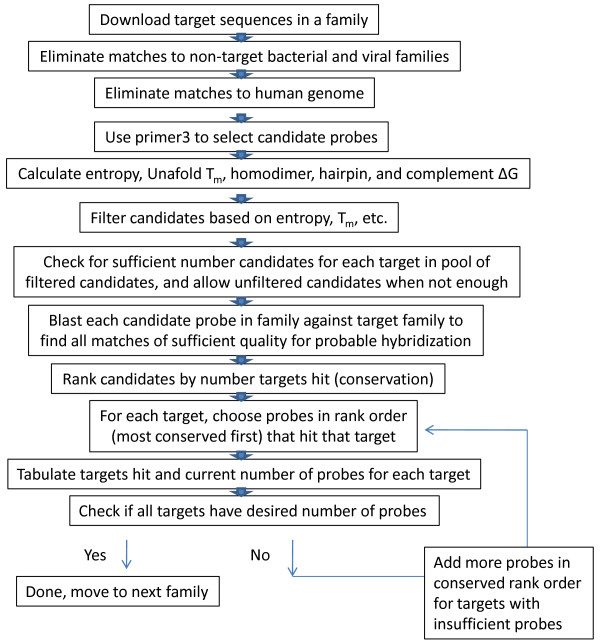
**Array design process diagram, illustrating the probe selection algorithm described in the text**.

The array design process is diagrammed in Figure 1. In designing probes for our array, we sought to balance the goals of conservation and uniqueness, prioritizing oligo sequences that were conserved, to the extent possible, within the family of the targeted organism, and unique relative to other families and kingdoms. The design process is detailed in Methods, and summarized here.

We designed arrays with larger numbers of probes per sequence (50 or more for viruses, 15 or more for bacteria) than previous arrays having only 2-10 probes per target. The large number of probes per target was expected to improve sensitivity, an important consideration given possible amplification bias in the random PCR sample preparation protocol, which could result in nonamplification of genome regions targeted by some probes [7]. All bacteria and viruses with sequenced genomes available at the time we began the MDA v.1 design (spring 2007) were represented: ~38,000 virus sequences representing ~2200 species, and ~3500 bacterial sequences representing ~900 species. Version 1 of the array had only viral probes. A second version of the array (MDA v.2) was designed using both viral and bacterial probes. Probes were selected to avoid sequences with high levels of similarity to human, bacterial and viral sequences not in the target family. Low levels of sequence similarity across families were allowed selectively, when the statistical model of probe hybridization used in our array analysis predicted a low likelihood of cross-hybridization.

Favoring more conserved probes within a family enabled us to minimize the total number of probes needed to cover all existing genomes with a high probe density per target, enhancing the capability to identify the species of known organisms and to detect unsequenced or emerging organisms. Strain or subtype identification was not a goal of probe design for this array. Nevertheless, our ability to combine information from multiple probes in our analysis made it possible to discriminate between strains of many organisms.

The array design also incorporated a set of 2,600 negative control probes. These probes had sequences that were randomly generated, but with length and GC content distributions chosen to match those of the target-specific probes.

### Modeling of probe-target hybridization

We developed a novel statistical method for detection array analysis, by modeling the likelihood of the observed probe intensities as a function of the combination of targets present in the sample, and performing greedy maximization to find a locally optimal set of targets; the details of the algorithm are shown in Methods. It incorporates a probabilistic model of probe-target hybridization based on probe-target similarity and probe sequence complexity, with parameters fitted to experimental data from samples with known genome sequences. To accurately determine the organism(s) responsible for a given array result, the pattern of both positive and negative probe signals is taken into account. The algorithm is designed to enable quantifiable predictions of likelihood for the presence of multiple organisms in a complex sample.

A key simplification used in this algorithm was to transform the probe intensities to binary signal values ("positive" or "negative"), representing whether or not the intensity exceeds an array-specific detection threshold. The threshold was typically calculated as the 99^th ^percentile of the intensities of the random control probes on the array. The outcome variables in the likelihood model are the positive signal probabilities for each probe, given the presence of a particular combination of targets in the sample. The resulting predictions are more robust in the presence of noisy data, since the outcome variable is a probability rather than the actual intensity. Discretizing the intensities also led to considerable savings of computation time and resources, which are significant for arrays containing hundreds of thousands of probes.

**Figure 2:**
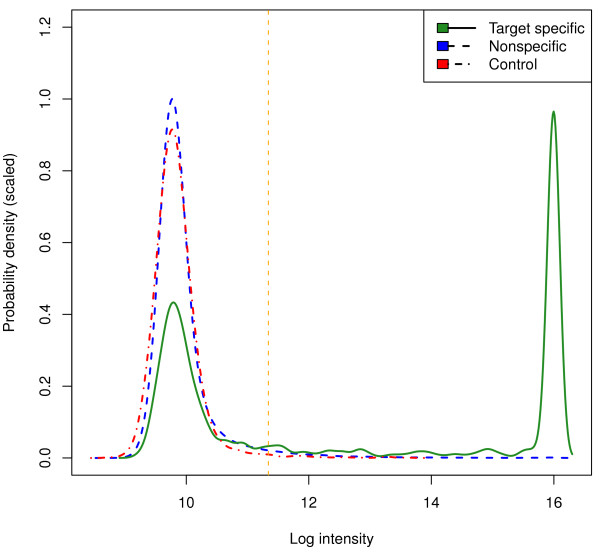
**Intensity distributions for an MDA v.2 array hybridized to a spiked mixture of vaccinia virus and HHV6B, for probes with and without target-specific BLAST hits and for negative control probes**. Vertical line: 99^th ^percentile of negative control distribution.

Although one might assume that reducing intensities to binary values means discarding valuable information, the log intensity distribution for a typical array (Figure 2) shows that the actual information loss is much less than expected. The figure shows separate density curves for three classes of probes: those with BLAST hits to one of the known targets in the sample ("target-specific"), those without hits ("nonspecific"), and negative controls. A vertical dashed line is drawn at the 99^th ^percentile threshold intensity. Log_2 _intensities for target-specific probes either cluster with the control and nonspecific probes (when they have low BLAST scores, usually), or approach the maximum possible value (16). This occurs because detection array probes are designed for high sensitivity to low target concentrations, so that probe intensities approach the saturation level whenever a probe has significant similarity to a target in the sample. Therefore, the information content of a probe signal is already reduced by saturation effects.

**Figure 3:**
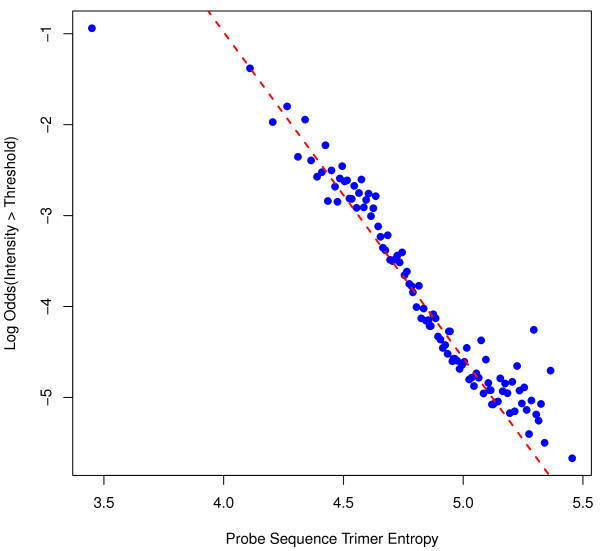
**Dependence of nonspecific positive signal frequency on the trimer entropy of the probe sequences**. Dashed line is a logistic regression fit to the probe entropy and signal data.

Certain probes were found to be more likely than others to yield positive signals, even when the sample on the array was known to lack any targets with sequences complementary to them. We observed that this nonspecific hybridization occurs more often with probes having low sequence complexity, i.e. long homopolymers and tandem repeats. One measure of the complexity of a probe sequence is the entropy of its trimer frequency distribution, which we compute as described in Methods. To study whether the sequence entropy could be used as a predictor of nonspecific hybridization, we selected data from nine MDA v2 arrays for which all sample components had known genome sequences. We selected probes with no BLAST hits to any of the known targets, grouped them by entropy into equal sized bins, computed the positive signal frequency (the fraction of probes with positive signals), converted the frequency to a log-odds value, and plotted the log-odds against the trimer entropy, as shown in Figure 3. We also fit a logistic regression model for the probe signal as a function of entropy; a dashed line with the resulting slope and intercept is shown in the plot. The figure shows that the trimer entropy is an excellent predictor of the non-specific positive signal probability, and that probes with low entropy are more likely to give positive signals regardless of the target sequence.

While the nonspecific probe signal probability depends on the probe sequence only, the target-specific signal probability was assumed to be a function of both the probe sequence and probe-target sequence similarity. To determine an appropriate set of predictors for the specific signal probability, given the presence of a specific target, we BLASTed the probe sequences against our database of target genomes, obtaining the best alignment (if any) for each probe-target pair. We then derived various covariates from the probe-target alignment, including the alignment length, number of mismatches, bit score, E-value, predicted melting temperature, and alignment start and end positions. We tested all combinations of up to three covariates, using logistic regression to fit models to data from samples containing known targets, and performed leave-one-out validation to find the combination with the strongest predictive value. The best combination included three covariates: (1) The predicted melting temperature, computed as described in Methods; (2) the BLAST bit score and (3) the alignment start position relative to the 5' end of the probe. We expected the alignment start position to have a significant effect, because we observed in our previous work [2] that probe-target mismatches had a weaker effect on hybridization if the mismatch was closer to the 3' end of the probe (nearer to the array surface).

### Likelihood maximization algorithm

To find a combination of targets whose presence in the sample best explains the observed data, we used a greedy algorithm to find a local maximum for the log-likelihood, as described in Methods. We can think of the likelihood maximization algorithm as an iterative process, in which we first find the target that explains the largest portion of the observed positive probe signals, while minimizing the number of negative probes that would be expected to bind to the target. In each subsequent iteration, we choose the target that explains the largest part of the signal not already explained by the first target, while again minimizing the number of expected negative probes. The process continues until a maximal portion of the observed probe signals are explained, or for a specified maximum number of iterations.

**Figure 4:**
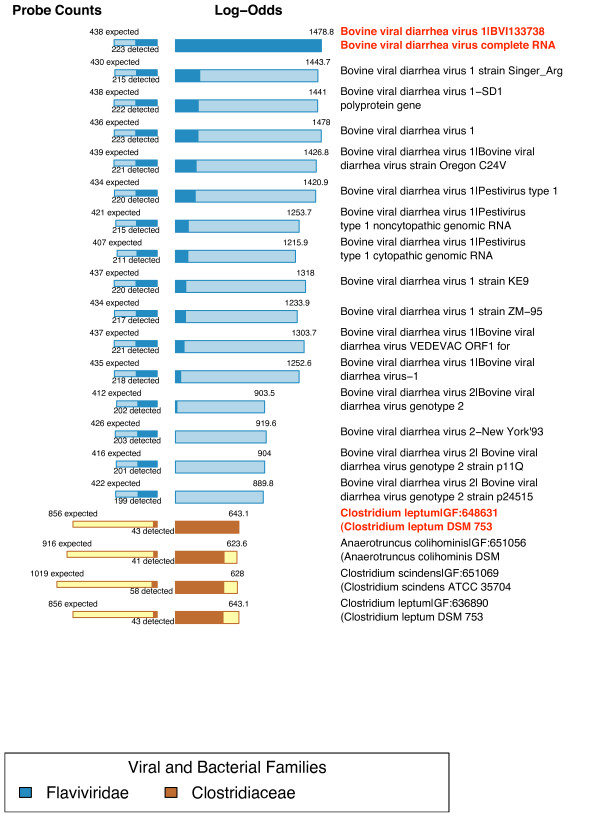
**MDA v2 results for a spiked sample of bovine viral diarrhea virus**. Many of the same conserved probes that hybridize to BVDV also match classical swine fever virus and Border disease virus, although these have a lower log-odds and so are not the "detected" organism, which is labeled in red.

**Figure 5:**
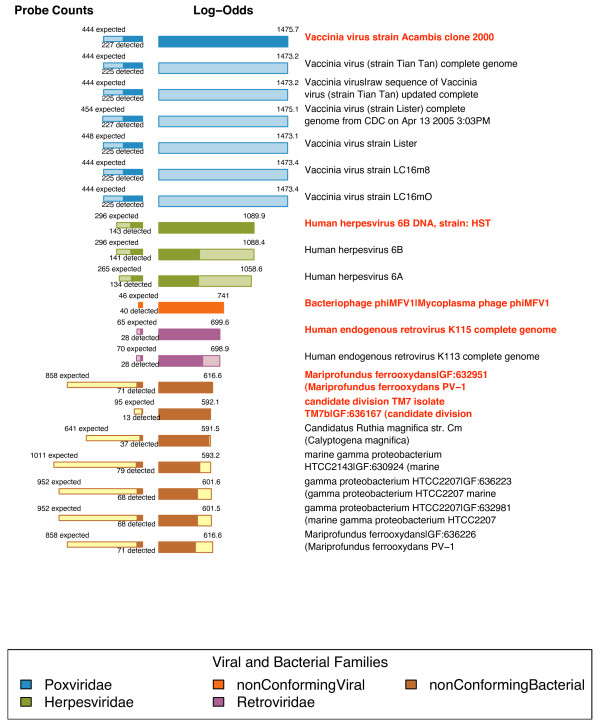
**MDA v.2 results for a spiked mixture of vaccinia virus and HHV6B**.

**Figure 6:**
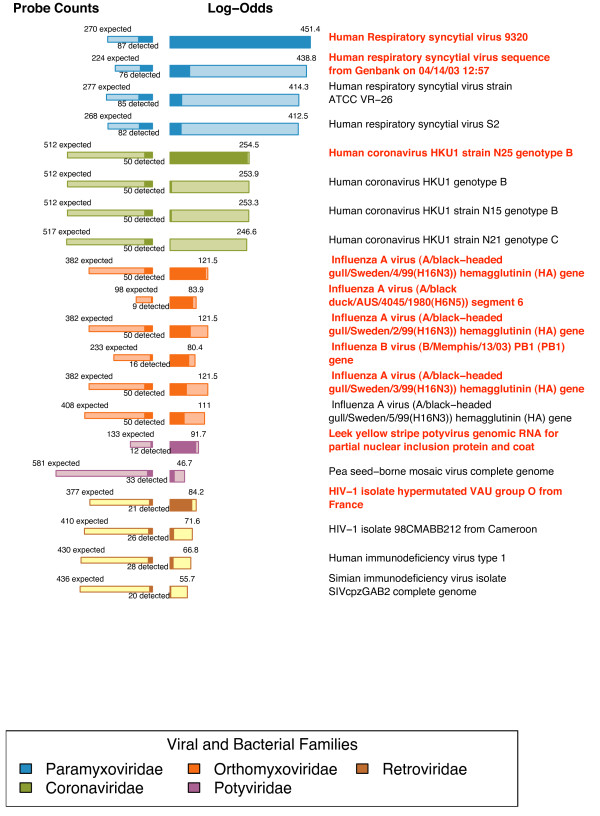
**MDA v.1 results for a clinical induced sputum sample**.

**Figure 7:**
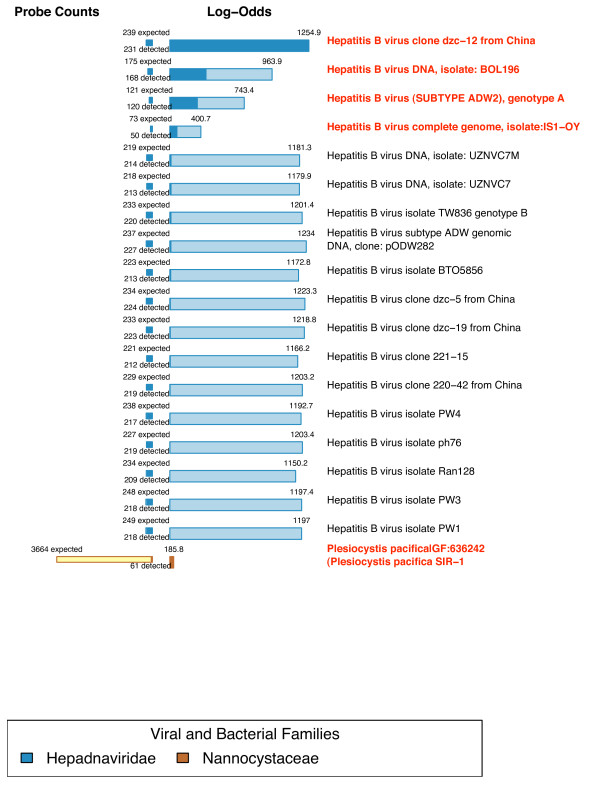
**Clinical serum sample 1_5, provided by the DeRisi lab at UCSF, analyzed on MDA v.2**.

**Figure 8:**
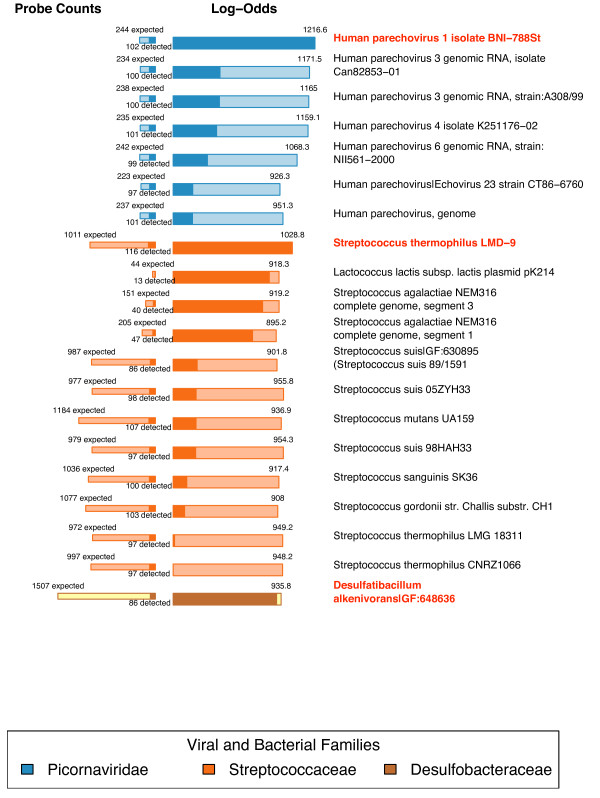
**Clinical fecal sample 2_1, provided by the DeRisi lab at UCSF, analyzed on MDA v.2**.

**Figure 9:**
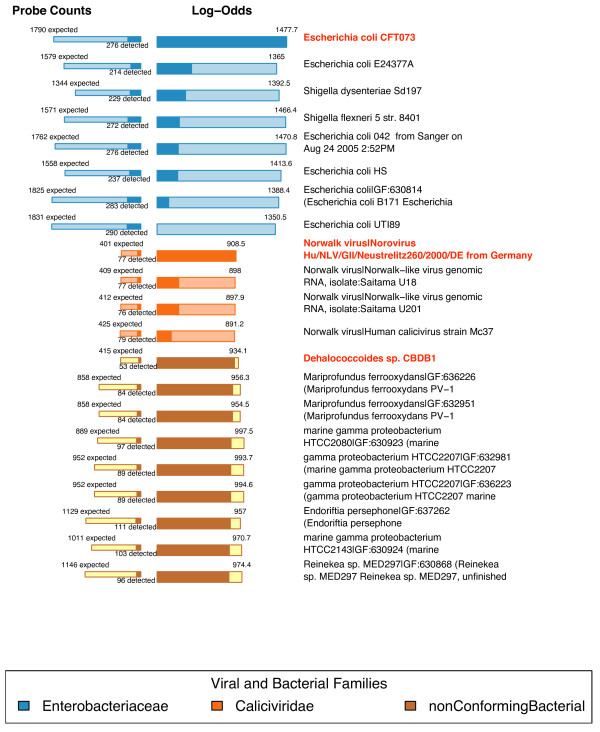
**Clinical fecal sample 2_2, provided by the DeRisi lab at UCSF, analyzed on MDA v.2**.

**Figure 10:**
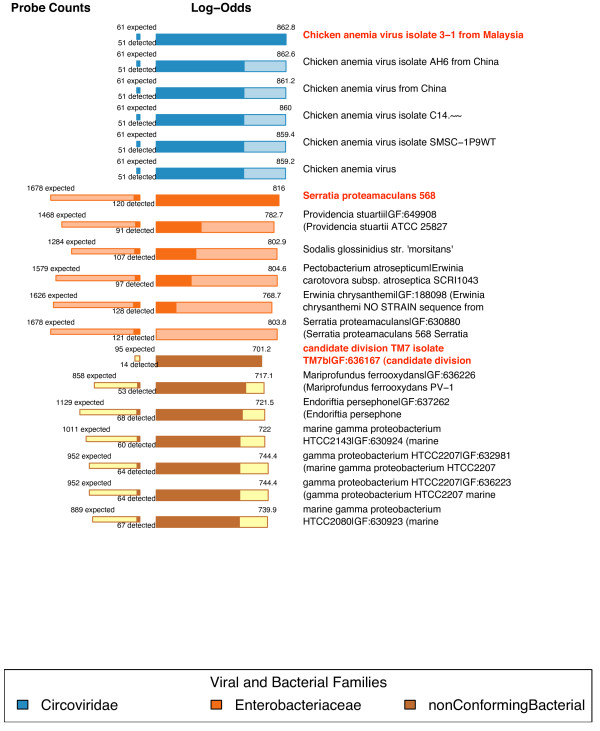
**Clinical fecal sample 2_3, provided by the DeRisi lab at UCSF, analyzed on MDA v.2**.

**Figure 11:**
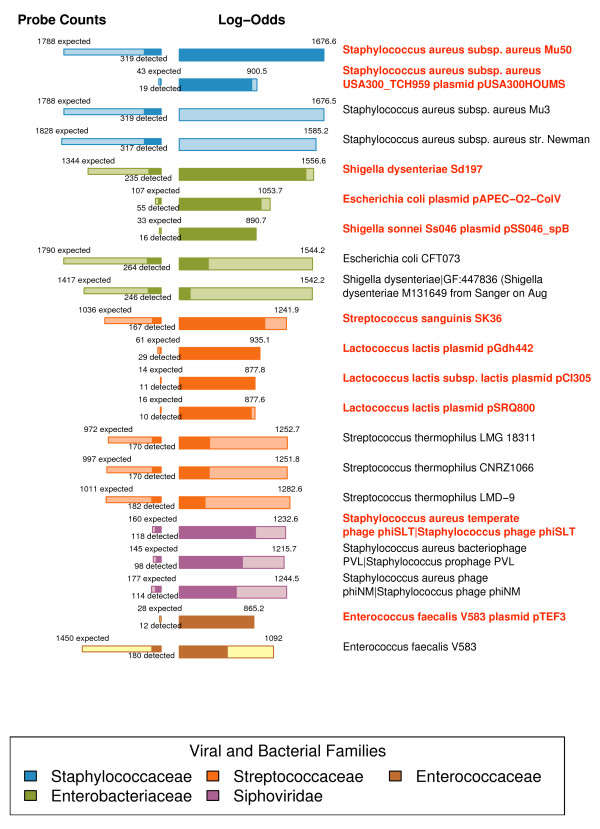
**Clinical fecal sample 2_4, provided by the DeRisi lab at UCSF, analyzed on MDA v.2**.

The analysis results are typically visualized as shown in Figures 4, 5, 6, 7, 8, 9, 10, and 11. The bar graphs in the right-hand column show the initial and final log-odds scores for the target genomes predicted to be present (annotated in red boldface), together with the highest-scoring targets in the same taxonomic families as the predicted targets. The bars are divided into light and dark shaded sections, corresponding to the initial and final scores respectively. Bars with the same hue correspond to targets in the same family.

The bar graphs in the left-hand column represent expectation (mean) values of the positive probe counts for each target, given the presence of the corresponding target genome. The larger "expected" score is obtained by summing the positive signal probabilities for all probes; the smaller "detected" score is derived by limiting this sum to probes having positive signals. Because probes often cross-hybridize to multiple related genome sequences, the numbers of "expected" and "detected" probes may greatly exceed the number of probes that were actually designed for a given target organism. The probe count bar graphs are designed to provide some additional guidance for interpreting the prediction results.

### Testing on pure and mixed samples of known viruses

Several viruses with sequenced genomes (adenovirus type 7, respiratory syncytial virus [RSV], and bovine viral diarrhea virus [BVDV]) were each hybridized to MDA v.1 arrays in separate array experiments. Analysis of each array identified the known virus as the highest scoring target (data not shown). Several mixtures of both RNA and DNA viruses were also tested (Table 1). All spiked species were detected in the mixture, including most of the segments of bluetongue virus (BTV). Strain discrimination was not expected, since probes were designed from regions conserved within viral families. Nevertheless, the highest scoring targets in the single virus experiments with adenovirus, BVDV, vaccinia and human herpesvirus 6B (HHV6B) were in fact the strains hybridized to the arrays. Human endogenous retrovirus K113 was also detected in two of the three mixtures, possibly from host cell DNA.

**Table 1:** Results of initial tests on MDA v.1

Mixture tested	Detected	Additionally detected
Adenovirus type 7 strain Gomen	Yes	Human endogenous retrovirus K113
Respiratory syncytial virus strain Long	Yes	Leek yellow stripe potyvirus
Bovine viral diarrhea virus type 1 strain Singer	Yes	

Respiratory syncytial virus strain B1	Yes	None
Bluetongue virus type 2	Yes (segments 2,6,8,9,10)	

Human herpesvirus 6B	Yes	Human endogenous retrovirus K113
Vaccinia virus strain Lister	Yes	Influenza A segment 8
Respiratory syncytial virus strain B1	Yes	
Bluetongue virus type 2	Yes (segments 2,6,7,8,9,10)	

For three samples tested, we compared the spiked strain identities with those predicted by analyzing either 1) only the LLNL probes versus 2) analyzing only the ViroChip probes which were also included on the MDA. The LLNL probes identified the correct Gomen strain of human adenovirus type 7 while the ViroChip probes identified the correct species but the incorrect NHRC 1315 strain. In another example, when RSV Long group A (an unsequenced strain) was hybridized to the array, the related RSV strain ATCC VR-26 was predicted by MDA probes, but the ViroChip probes failed to detect any RSV strain. For the detection of BVDV Singer strain, both LLNL and ViroChip probes were able to predict the exact strain hybridized.

False negative error rates were estimated for MDA v.1 from experiments in which some or all of the viruses in the sample had known genome sequences (Table 2), for probes that met our design criteria (85% identity and a 29 nt perfect match to one of the target genome sequences). The RSV and BTV probes were excluded from this estimate, as sequences were not available for the exact strains used in the experiments. All 128 selected probes had signals above the 99^th ^percentile detection threshold, yielding a zero false negative error rate.

**Table 2:** True positive/false negative counts for probes in MDA v.1 tests with sequenced viruses

Target	Number of perfect match probes	TP probes	FN probes	Percent FN error rate
**Pure viral cultures:**				
Adenovirus type 7 Gomen	52	52	0	0.0
Bovine viral diarrhea virus (BVDV)	25	25	0	0.0
				
**Mixture of viral cultures:**				
Human herpesvirus 6B	14	14	0	0.0
Vaccinia virus Lister strain	37	37	0	0.0
**Total**	**51**	**51**	**0**	**0.0%**
				
**Overall**	**128**	**128**	**0**	**0.0%**

To validate MDA v.2 with known spiked viruses, BVDV type 1 (Figure 4) and a mixture of vaccinia Lister and HHV 6B (Figure 5) were correctly identified to the species level. Virus sequences selected as likely to be present are highlighted in red in these figures. On the vaccinia+HHV 6B array, human endogenous retrovirus K113 was also detected. In addition, several organisms that were unlikely to be present were predicted, probably because of non-specific probe binding or cross-hybridization. These organisms, *Mariprofundus ferrooxydans *(a deep sea bacterium collected near Hawaii), candidate division TM7 (collected from a subgingival plaque in the human mouth), and marine gamma-proteobacterium (collected in the coastal Pacific Ocean at 10 m depth) were detected with low log-odds scores on numerous experiments using different samples. Genome sequences for these were not included in the probe design because they became available only after we designed the microarray probes or because they were not classified into a bacterial taxonomic family; therefore probes were not screened for cross-hybridization against these targets. However, these sequences were included in the analysis database, since this database included sequences not classified as to bacterial family and it was compiled a few months after the probes were designed. Genome comparisons indicate that *M. ferrooxydans*, TM7b, and marine gamma proteobacterium HTCC2143 share 70%, 55%, and 61%, respectively, of their sequence with other bacteria and viruses, based on simply considering every oligo of size at least 18 nt also present in other sequenced viruses or bacteria, so many of the probes designed for other organisms may also hybridize to these targets.

### Testing on Blinded Samples from Pure Culture

Blinded samples were provided from collaborator Robert Tesh at UTMB for 11 viruses. We hybridized each of those samples separately to MDA v.2 and predicted the identities of each virus (Table 3). Ten of 11 of the species predicted by the MDA were confirmed by Dr. Tesh. In addition, endogenous retroviruses were also detected in 7 of the samples as well as the uninfected Vero cell control, indicating the presence of host DNA from the culture cells. These included one or more of the following: Baboon endogenous virus strain M7 and human endogenous retroviruses K113, K115, and HCML-ARV, with human endogenous retrovirus K113 being the most common. The one sample that we failed to detect on the array was vesicular stomatitis virus, NJ (VSV NJ). We confirmed that it was present in the sample using two proprietary, unpublished TaqMan assays developed by colleagues at LLNL and tested by LLNL colleagues at Plum Island that specifically detect VSV NJ. There were no complete genomes of VSV NJ available. Consequently, we designed no probes for this species. Nor was it included in our database for the statistical analyses. It is sufficiently different from the genomes available for VSV Indiana that none of those probes had BLAST similarity to the partial sequences available for VSV NJ. There were 7 probes from the ViroChip corresponding to VSV NJ that were detected. These probes were designed from partial sequences [4].

**Table 3:** Results of blinded testing on viral isolate samples from Robert Tesh at University of Texas, Medical Branch

ID	Culture results	Array results
---	Vero cells not infected	Background signal
TVP-11180	Punta Toro	Punta Toro virus strain Adames
TVP-11181	Thogoto	Thogoto virus strain IIA
TVP-11182	Dengue 4	Dengue 4 strain ThD4_0734_00
TVP-11183	CTF	Colorado tick fever virus
TVP-11184	Cache Valley	Cache Valley genome RNA for N and NSs proteins
TVP-11185	Ilheus	Ilheus virus
TVP-11186	EHD-NJ	Epizootic hemorrhagic disease virus isolate 1999_MS-B NS3
TVP-11187	La Crosse	La Crosse virus strain LACV
TVP-11188	SF Sicilian	Sandfly fever Sicilian virus
TVP-11189	VSV-NJ	Not detected
TVP-11191	Ross River	Ross River virus

### Detection of viruses and bacteria from clinical samples

We tested a clinical induced sputum sample provided to us by the UCSF DeRisi lab on MDA v.1 (Figure 6), and detected human respiratory syncytial virus and human coronavirus HKU1. We confirmed the results using specific PCR for these two viruses (Additional file 1). The results were also confirmed by the DeRisi lab using the ViroChip. The MDA results indicated small log-odds scores for influenza A, leek yellow stripe potyvirus, and HIV-1, although these low scores are a result of just a few probes and are likely due to nonspecific binding rather than true positives. Other samples tested using MDA v.1 also had low scoring predictions for Influenza A and Leek yellow stripe potyvirus (see Table 1), which we suspect were due to non-specific binding.

Closer examination of probes giving high signal intensities that were not consistent with the "detected" organisms showed that there were some "sticky" probes that seemed to bind non-specifically. On the MDA v.2 array, we noted that 141 probes were detected in a majority (31 out of 60) of arrays hybridized to a wide variety of sample types. A small number of these probes were found to have significant BLAST hits to the human genome. Since most of the samples tested on the array were either human clinical samples or were grown in Vero cells (an African green monkey cell line), the frequent high signals for these few probes can be explained by the presence of primate DNA in the sample. The vast majority of sticky probes, however, were not explained by cross-hybridization to host DNA. We did note significant differences between sticky and non-sticky probes in the distributions of trimer entropy and hybridization free energy; sticky probes had smaller entropies (mean 4.6 vs 4.8 bits, *p = 7.5 *× 10 ^-14^) and more negative free energies (mean -70.5 vs -66.8 kcal/mol, *p *= 3.8 × 10 ^-13^) compared to 1755 non-sticky probes detected in 11 or fewer samples. Consequently, in v.2 of the chip design, we imposed an entropy filter as described in the methods, and additionally designed more probe sequences at the expense of the number of replicates per probe.

We tested partially amplified clinical samples provided by Joe DeRisi's laboratory at UCSF on MDA v.2. The source (e.g. fecal or serum) was blinded during our analyses, but was provided later: sample 1_5 from serum (Figure 7), and the rest from fecal sources (Figures 8, 9, 10, and 11). No patient history was provided. A strong signal indicating the presence of hepatitis B virus was detected in sample 1_5 (Figure 7). In all the remaining samples, signals were detected for a mixture of both viruses and bacteria, many of which are known to be associated with gastrointestinal distress. All of the organisms detected by the array were confirmed by PCR except *Serratia proteamaculans *in sample 2_3 and the *E. coli *pAPEC O2-ColV plasmid in sample 2_4 (Additional file 1) In some cases, multiple species in a genus have similarly high log-odds ratios, suggesting that a member of that genus is likely to be present, but the MDA cannot make a definitive call as to which species. For example, signals for multiple *Streptococcus *species were detected in sample 2_1 (Figure 8). We expect that this pattern could arise in cases where a novel unsequenced isolate from the genus is present, in cases where conservation at the genus level is sufficiently high that the conserved probes do not provide species discrimination, or when multiple related organisms are present. Since the probes were selected from conserved regions within a family, the array was not designed for stringent species or strain discrimination. In sample 2_3 (Figure 10) we saw a strong signal for the presence of chicken anemia virus which was confirmed by PCR. This circovirus infects chickens, pointing to a dietary influence on the components of this human fecal sample. It is similar to the recently discovered and widespread human TT virus and TTV-like mini virus (TLMV) [21]. Other studies have also found viruses from food in fecal samples [22]. Phage are also commonly detected in feces, particularly when sample preparation enriches for phage, for example, by purification in a cesium chloride gradient [23]. We did not do any gradient purification for phage enrichment, and although we detected more signals from bacteria than from the bacteriophage that infect those bacteria, we did detect signals for *Staphylococcus aureus *phage as the viral component of sample 2_4, as well as signals that its host bacteria was present (Figure 11).

## Discussion

We report on a high density oligo microarray and statistical analysis method that has detected viral and bacterial sequences from single DNA and RNA viruses and mixtures thereof, various clinical samples, and blinded cell culture samples provided to us by our collaborators. Results from clinical samples were validated using PCR. The MDA v.2 has higher probe density and larger phylogenetic representation of viral and bacterial sequenced genomes than other published array designs. It can be applied to problems in viral and bacterial detection from pure or complex environmental or clinical samples. It may be particularly useful to widen the scope of search for microbial identification when specific PCR fails, as well as to identify co-infecting organisms.

The analysis method described here differs in several key ways from previous statistical approaches to analyze microbial detection arrays, such as E-Predict [24] and GreeneLAMP [12]. E-Predict compares a probe intensity vector for each array against a theoretical hybridization free energy vector for each sequence in a target database, both normalized to unit length, using the Pearson correlation coefficient. Significance values are assigned to the correlation scores by comparing them against an empirical distribution derived from over 1,000 array experiments. To handle samples that may contain a mixture of organisms, there is an iterative version of E-Predict, which, after identifying the most likely target on an array, sets the intensities of the probes matching the target to zero, computes new correlation coefficients, and repeats the identification process. Since it requires a corpus of data from previous experiments with the same array design, E-Predict cannot be applied to new, prototype array designs. E-Predict also has limited applicability to complex samples, in which several closely related strains of the same species may be present.

The GreeneLAMP algorithm [12] assigns probes to target taxa using BLAST alignments, and computes a *p*-value for each probe intensity by performing a *z*-test against a log-normal distribution. Intensities are first background-corrected, using data from a matched control array when available, or random control probes on the same array otherwise. The parameters of the distribution are inferred from the intensities of all probes on the array, under the assumption (usually justified) that only a small fraction of probes will hybridize to the target on the array. Probes are categorized as positive or negative by comparing their *p*-values against a fixed threshold. The *p*-values for the positive probes associated with each taxon are then multiplied, and an aggregate *p*-value computed for the product, using the method of Bailey [25]. Finally, candidate taxa are ranked by the aggregate *p*-value. The GreeneLAMP method depends heavily on the assumption that probe intensities are independent measurements of target genome abundances, which is not always justified. An iterative version of the method has not been reported to date; this makes it inappropriate for identifying mixtures of organisms in a sample, since the output of the algorithm is a single ranked list of taxa

The MDA array design and accompanying analysis algorithm have been found to perform well in identifying mixtures of known pathogens. In cases where a sample contains an organism that has not been sequenced (or whose sequence is not in our analysis database), but is sufficiently similar to other sequenced microbes, the analysis will identify multiple related organisms most similar to the one in the sample. Similar results will be seen when the sample DNA is degraded or low in concentration, so the analysis cannot determine that a novel or unsequenced organism is present. Therefore, users of the MDA will need to interpret the data in the context of what else is known about the sample, to determine whether the predicted organisms are exact matches to known species or are novel but with some similarity to other sequenced microbes. Highly novel targets with no similarity to genomes in the database or probes on the array will not be detected. The failure of the MDA to detect VSV NJ in pure culture illustrates this well, and highlights a shortcoming of our approach to use only complete genomes to design the array. Future versions of the array will include all available sequence data, including partial sequences and gene fragments, for species lacking complete genomes. Modified statistical algorithms will then be required to deal with sequence length bias when partial genomes are included in the database. Most importantly, as new sequence data becomes available from newly discovered or newly sequenced organisms, the MDA must be updated with probes to detect them. We are currently designing Version 3 of the MDA to address these issues, probing both partial and complete bacterial and viral sequences, as well as fungi and some protozoa.

## Conclusions

The MDA is a tool to identify viral and bacterial organisms present in simple or complex samples. We have demonstrated the capability of the array and our statistical methods to identify multiple bacteria and viruses in clinical samples and verified results with PCR. Improvements are suggested for the design of future detection arrays, and continued updates to incorporate probes for newly sequenced microbes will be required.

## Methods

### Probe design for family level characterization

Figure 1 summarizes our array design process. We downloaded all complete genomes, segments, and plasmid sequences, organized by family, for all bacteria and viruses, from NCBI Genbank, Integrated Microbial Genomics (IMG) project at the Joint Genome Institute, The Comprehensive Microbial Resource (CMR) at the JC Venter Institute, and The Sanger Institute in the United Kingdom, with some additional proprietary whole-genome data from collaborators. We included only bacteria under the superkingdom Bacteria (eubacteria) taxonomy node at NCBI, and did not include the Archaea. Sequence data was current as of the time that probe design began for a given probe set: 34,625 viral target sequences on April 25, 2007 (viral probes, v.1), 38,402 viral target sequences on October 29, 2007 (viral probes, v.2), and 3,477 bacterial target sequences on July 12, 2007 (bacterial probes, v.2). These represented 2195 viral species and 924 bacterial species.

Probes were selected from whole genomes, without regard to gene locations or identities, letting the sequences themselves determine the best signature regions and preclude bias by pre-selection of genes. In prior work, we have found that the length of longest perfect match (PM) is a strong predictor of hybridization intensity, and that for probes at least 50 nt long, PM ≤ 20 bp have signal less than 20% of that with a perfect match over the entire length of the probe.[2] This is similar to results from a systematic study of viral probe hybridization characteristics by [7]. Therefore, for each target family we eliminated regions with perfect matches to sequences outside the target family. Using the suffix array software Vmatch [26], perfect match subsequences of at least 17 nt long present in non-target viral families or 25 nt long present in the human genome or nontarget bacterial families were eliminated from consideration as possible probe subsequences. Sequence similarity of probes to non-target sequences below this threshold was allowed, but could be accounted for using the statistical algorithm described below.

From these family-specific regions, we designed probes 50-66 bases long for one family at a time using the methods described in [2]. Probes were sufficiently long (50-66 bases) to tolerate some sequence variation, although slightly shorter than the 70-mer probes used on previous arrays [4,11,12] because of the additional synthesis cycles, and therefore cost, of making 70-mers on the NimbleGen platform. Long probes improve hybridization sensitivity and efficiency, alleviate sequence-dependent variation in hybridization, and improve the capability to detect unsequenced microbes. Quantifying the microbe load was not a goal of this array; sensitive detection of microbe presence was the aim, facilitated by the higher sensitivity of longer probes.

As in [2], we generated candidate probes using MIT's Primer3 software [27], followed by T_m _and homodimer, hairpin, and probe-target free energy (ΔG) prediction using Unafold [28]. Candidate probes with unsuitable ΔG's or T_m_'s were excluded as described in [2]. Desirable range for these parameters was 50 ≤ length ≤ 66, T_m_≥80°C, 25% ≤ GC% ≤ 75%, ΔG_homodimer _= ΔG of homodimer formation > 15 kcal/mol, ΔG_hairpin _= ΔG of hairpin formation > -11 kcal/mol, and ΔG_adjusted _= Δ*G_complement _- 1.45 *Δ*G_hairpin _- 0.33 *Δ*G_homodimer _*≤ -52 kcal/mol. For the v2 array design, which includes bacterial probes, an additional minimum sequence complexity constraint was enforced, requiring a trimer frequency entropy of at least 4.5 (calculation described below). If fewer than a minimum number of candidate probes per target sequence passed all the criteria, then those criteria were relaxed to allow a sufficient number of probes per target. To relax the criteria, first candidates that passed the Primer3 criteria but failed the Unafold filters were allowed. If no candidates passed the Primer3 criteria, then regions passing the target-specificity (e.g. family specific) and minimum length constraints were allowed. From these candidates, we selected probes in decreasing order of the number of targets represented by that probe (i.e. probes detecting more targets in the family were chosen preferentially over those that detected fewer targets in the family), where a target was considered to be represented if a probe matched it with at least 85% sequence similarity over the total probe length, and a perfectly matching subsequence of at least 29 contiguous bases spanned the middle of the probe. For probes that tied in the number of targets represented, a secondary ranking was used to favor probes most dispersed across the target from those probes which had already been selected to represent that target. The probe with the same conservation rank that occurred at the farthest distance from any probe already selected from the target sequence was the next probe to be chosen to represent that target.

The MDA v.1 array contained probes representing all complete viral genomes or segments associated with a known viral family, with at least 15 probes per target (Table 4). It did not include unclassified targets not designated under a family. On the v.2 array, every viral genome or segment was represented by at least 50 probes, totaling 170,399 probes, except for 1,084 viral genomes that were not associated under a family-ranked taxonomic node ("nonConforming sequences"). These had a minimum of 40 probes per sequence totaling 12,342 probes. There were a minimum of 15 probes per bacterial genome or plasmid sequence, totaling 7,864 probes on the v.2 array. Bacterial genomes that were not associated under a family-ranked taxonomic node were not included in the array design.

**Table 4:** Summary of array design: Probe counts

Number of Probes	Probe Description
Version 1	
36497	Viral detection probes (15 probes/seq from each taxonomic family)
20736	ViroChip probes
1278	human viral response genes
3000	random controls
Version 2	
170399	Viral probes (50 probes/seq from each taxonomic family) × 2 replicates
12342	nonConforming viruses (not associated w/ taxonomic family, 40 probes/seq)
7864	bacterial probes (15/seq)
20736	ViroChip probes
1278	human viral response genes
2651	random controls

On both MDA v.1 and v.2, as controls for the presence of human DNA/mRNA from clinical samples, we designed 1,278 probes to human immune response genes. For targets, the genes for GO:0009615 ("response to virus") were downloaded from the Gene Ontology AmiGO website http://amigo.geneontology.org, filtering for *Homo sapien *sequences. There were 58 protein sequences available at the time (July 12, 2007), and from these, the gene sequences of length up to 4× the protein length were downloaded from the NCBI nucleotide database based on the EMBL ID number, resulting in 187 gene sequences. Fifteen probes per sequence were designed for these using the same specifications as for the bacterial and viral target probes.

We designed ~2,600 random control sequences that were length and GC% matched to the target probes on MDA v.1 or v.2. These had no appreciable homology to known sequences based on BLAST similarity, and were used to assess background hybridization intensity. These were designed by calculating the fraction f(L, g) of detection probes with length = L and GC% = g, and simulating f(L, g) times the number of random probes desired random sequences of length L and GC% g for each L, g observed in the detection probes. In addition, we also included on the v.1 and v.2 arrays the 21,888 probes from the ViroChip version 3 from University of California San Francisco [4,14,24,29] downloaded from http://www.ncbi.nlm.nih.gov/geo/query/acc.cgi?acc=GPL3429. We note that additional probe sets may be added to meet unique needs of specific customers, not discussed further here.

### Sample preparation and microarray hybridization

DNA microarrays were synthesized using the NimbleGen Maskless Array Synthesizer at Lawrence Livermore National Laboratory as described [2]. Adenovirus type 7 strain Gomen (Adenoviridae), respiratory syncytial virus (RSV) strain Long (Paramyxoviridae), respiratory syncytial virus strain B1, bluetongue virus (BTV) type 2 (Reoviridae) and bovine viral diarrhea virus (BVDV) strain Singer (Flaviviridae) were purchased from the National Veterinary lab and grown at our laboratory. Purified DNA from human herpesvirus 6B (HHV6B) (Herpesviridae) and vaccinia virus strain Lister (Poxviridae) were purchased from Advanced Biotechnologies (Maryland, VA). 11 blinded viral culture samples were received from Dr. Robert Tesh's lab at University of Texas Medical Branch at Galveston (UTMB). The viral cultures were sent to LLNL in the presence of Trizol reagent.

After treatment with Trizol reagent, RNA from cells was precipitated with isopropanol and washed with 70% ethanol. The RNA pellet was dried and reconstituted with RNase free water. 1 μg of RNA was transcribed into double-strand cDNA with random hexamers using Superscript™ double-stranded cDNA synthesis kit from Invitrogen (Carlsbad, CA). The DNA or cDNA was labeled using Cy-3 labeled nonamers from Trilink Biotechnologies and 4 μg of labeled sample was hybridized to the microarray for 16 hours as previously described (Jaing et al., 2008). Clinical samples that had been extracted and partially purified using Round A and Round B protocols (Wang et al, 2003) were obtained from Dr. Joseph DeRisi's laboratory at University of California, San Francisco (UCSF). The samples were amplified for an additional 15 cycles to incorporate aminoallyl-dUTP and labeled with Cy3NHS ester (GE Healthcare, Piscataway, NJ). The labeled samples were hybridized to NimbleGen arrays.

Data have being submitted to the Gene Expression Omnibus (GEO) database http://www.ncbi.nlm.nih.gov/geo/ accession number GSE24700.

### PCR for confirmation

Clinical samples from the DeRisi laboratory were tested by PCR to confirm the microarray results. PCR primers were designed using either the KPATH system [30] or based on the probes that gave a positive signal for the organism identified as present, and the primer sequences are proved as Additional file 1. PCR primers were synthesized by Biosearch Technologies Inc (Novato, CA). 1 μL of Round B material was re-amplified for 25 cycles and 2 μL of the PCR product was used in a subsequent PCR reaction containing Platinum Taq polymerase (Invitrogen), 200 mM primers for 35 cycles. The PCR condition is as follows: 96°C, 17 sec, 60°C, 30 sec and 72°C, 40 sec. The PCR products were visualized by running on a 3% agarose gel in the presence of ethidium bromide.

### Statistical analysis

For each array hybridization, we transformed the probe intensities *I_ik _*(for probe *i *on an array hybridized to sample *k*) to binary values *Y_ik_*, representing whether *I_ik _*exceeds an array-specific detection threshold. The threshold was typically calculated as the 99^th ^percentile of the intensities of the negative control probes.

We developed simple logistic models to predict two conditional probabilities: the probability of observing *Y_ik _*= 1 given the presence of a specific microbial target in sample *k*, and the probability of observing *Y_ik _*= 1 given no complementary targets. The predictors for the specific probability *P*(*Y_ik _*= 1 | target *j *is present) were derived by BLASTing probe *i*'s sequence against target *j*'s genome, with an E-value threshold of 0.1, and choosing the highest scoring alignment for the probe-target pair. The BLAST bit score *B_ij _*and the probe alignment start position *Q_ij _*were extracted directly from the BLAST output; the melting temperature *T_ij _*was computed according to the formula *T_ij _*= 69.4°C + (41 *N_GC _*- 600)/*L*, where *L *is the length of the alignment and *N_GC _*is the number of G or C bases in the alignment.

The entropy *S_i _*of the probe sequence trimer distribution was computed by counting the numbers of occurrences *n_AAA_, n_AAC_, ..., n_TTT _*of the 64 possible trimers in the probe sequence, and dividing by the total number of trimers, yielding the corresponding frequencies *f_AAA_, ..., f_TTT_*. The entropy is then given by:

$$S_{i}=\sum_{t:f_{t}\neq 0}-f_{t}{\text{ log}}_{\text{2}}f_{t}$$

where the sum is over the trimers *t *with *f_t _≠ *0. We estimate the nonspecific signal probability with a logistic model:

$$\begin{array}{l} P(Y_{ik}=1|\;\text{no target present})\\ =\frac{1}{1+\exp[-(a_{0}+a_{1}S_{i})]} \end{array}$$

We then model the specific probe signal probability by a logistic function in which the linear predictor combines both nonspecific and target-specific terms:

$$\begin{array}{l} P(Y_{ik}=1|\text{target }j\text{ present})\\ =\frac{1}{1+\exp[-(a_{0}+a_{1}S_{j}+a_{2}T_{ij}+a_{3}B_{ij}+a_{4}Q_{ij})]} \end{array}$$

To fit the parameters *a_0 _*through *a_4 _*in the above models, we ran array experiments using samples of viruses and bacteria with known genome sequences, computed the covariates *S_i_, T_ij_, B_ij_*, and *Q_ij_*, and performed logistic regression against the observed outcomes.

To apply this model to samples containing unknown targets, we performed an exhaustive BLAST search for every probe on the array against a comprehensive database of complete microbial genome sequences. We then computed the covariates *B_ij_, T_ij_*, and *Q_ij _*for each target on the array, for all probes having significant BLAST hits (E-value < 0.1) against the target.

The conditional probe signal probabilities are then combined to compute a likelihood function for the presence of a particular target, given the observed probe signals on an array. In the likelihood function, we assume that the probe signals are independent of one another, conditioned on the sample composition. Let *X *= (*X_1_, X_2_, ..., X_m_*) be a vector of target presence indicators, where *X_j _*= 1 if target *j *is present and 0 if not. The conditional likelihood of *X_j _*given the observed data *Y *can then be written:

(4) $$\begin{array}{l} L(X_{j};Y)=\\ \prod_{i\;:Yi=1}P(Yi=1|Xj)\prod_{i\;:Yi=0}P(Yi=0|Xj) \end{array}$$

where the individual probe-target signal probabilities are given by:

(5) $$\begin{array}{l} P(Yi=1|Xj)=\\ \frac{1}{1+\;\text{​}e^{-(a_{0}+a_{1}S_{i}+X_{j}(a_{2}T_{ij}+a_{3}B_{ij}+a_{4}Q_{ij}))}} \end{array}$$

When multiple targets may be present, an approximation is used to compute the probe signal probabilities:

(6) P(Yi=1|X)≈ 1−∏j:Xj=1P(Yi=0|Xj=1)=  1 −∏j:Xj=1 11+ea0+a1Si+a2Tij+a3Bij+a4Qij

Here we assume that the probability of obtaining a negative signal for a probe depends only on the set of targets that are assumed to be present, and that we can estimate the probability by multiplying the probabilities conditioned on the presence of the individual targets.

To find a combination of target presence indicators *X *that best explains the observed data, we use a greedy algorithm to find a local maximum for the log-likelihood. The algorithm is initialized by placing all candidate targets in an "unselected" list *U*, and creating an empty "selected" list *S*. The following steps are then iterated until the algorithm terminates:

*1*. Compute the conditional log-odds score *λ_j _*for each target *j *∈ *U:*

$$\begin{array}{l} \lambda_{j}=\sum_{i:Y_{i}=1}\text{log }\frac{P(Y_{i}=1|X_{j}=1,\;X_{k}=1\;\forall k\in S)}{P(Y_{i}=1|X_{j}=0,\;X_{k}=1\;\forall k\in S)}\\ +\sum_{i:Y_{i}=0}\text{log }\frac{P(Y_{i}=0|X_{j}=1,\;X_{k}=1\;\forall k\in S)}{P(Y_{i}=0|X_{j}=0,\;X_{k}=1\;\forall k\in S)} \end{array}$$

*λ_j _*is the log of the ratio of the likelihood of the data, if target *j *is added to *S*, to its likelihood if *j *is not added. When this step is performed for the first time, the selected list *S *will be empty, so the computed log-odds score for target *j *will not be conditioned on the presence of any other targets. We store this "initial" log-odds score *λ_j_^(i) ^*for later display.

2. Choose the target *j *that yields the largest value of *λ_j_*, remove it from list *U*, and add it to the end of list *S*. We store the value of this "final" score *λ_j_^(f) ^*for each target in *S*.

3. Repeat steps 1 and 2 until there is no target in *U *that yields a positive value for the conditional log-odds score (i.e., that increases the log-likelihood).

The result of this analysis is an ordered series *S *of target genomes predicted to be present, together with a pair of scores for each target in *S*. The initial score *λ_j_^(i) ^*is its log-odds from the first iteration; that is, the log of the ratio of the likelihood with target *j *present to the likelihood with no targets present. The final score *λ_j_^(f) ^*is the contribution of target *j *to the log likelihood at the time that it was selected, assuming the presence of all the targets that were selected prior to *j*.

The analysis algorithm is implemented in the Python language, except for plotting which is performed using the R programming environment. The software is available on request from the authors.

## Competing interests

There is a patent pending by the authors related to the MDA array design and analysis methods. We are employees of Lawrence Livermore National Security, LLC. LLNS, LLC manages the Lawrence Livermore National Laboratory for the Department of Energy under the contract DE-AC52-07NA27344.

## Authors' contributions

All authors conceived of the work. SNG designed the arrays and wrote the paper, CJJ performed the laboratory experiments and contributed to writing the paper, KSM designed and performed the statistical analyses and contributed to writing the paper, and TRS provided critical guidance and ideas for all parts of the work. All authors read and approved the manuscript.

## Supplementary Material

Additional file 1 **Primer sequences and product sizes used to confirm the array results from clinical samples**. Click here for file

## References

[B1] Nakamura S Yang C-S Sakon N Ueda M Tougan T Yamashita A Goto N Takahashi K Yasunaga T Ikuta K Direct Metagenomic Detection of Viral Pathogens in Nasal and Fecal Specimens Using an Unbiased High-Throughput Sequencing Approach PLoS ONE 2009 4 1 e4219 10.1371/journal.pone.0004219 19156205 PMC2625441

[B2] Jaing C Gardner S McLoughlin K Mulakken N Alegria-Hartman M Banda P Williams P Gu P Wagner M Manohar C A Functional Gene Array for Detection of Bacterial Virulence Elements PLoS ONE 2008 3 5 e2163 10.1371/journal.pone.0002163 18478124 PMC2367441

[B3] Lin B Blaney KM Malanoski AP Ligler AG Schnur JM Metzgar D Russell KL Stenger DA Using a Resequencing Microarray as a Multiple Respiratory Pathogen Detection Assay J Clin Microbiol 2007 45 2 443 452 10.1128/JCM.01870-06 17135438 PMC1829030

[B4] Wang D Urisman A Liu Y Springer M Ksiazek T Erdman D Mardis E Hickenbotham M Magrini V Eldred J Viral Discovery and Sequence Recovery Using DNA Microarrays PLoS Biol 2003 1 2 e2 10.1371/journal.pbio.0000002 14624234 PMC261870

[B5] Rota PA Oberste MS Monroe SS Nix WA Campagnoli R Icenogle JP Penaranda S Bankamp B Maher K Chen M-h Characterization of a Novel Coronavirus Associated with Severe Acute Respiratory Syndrome Science 2003 300 5624 1394 1399 10.1126/science.1085952 12730500

[B6] Urisman A Molinaro RJ Fischer N Plummer SJ Casey G Klein EA Malathi K Magi-Galluzzi C Tubbs RR Ganem D Identification of a Novel Gammaretrovirus in Prostate Tumors of Patients Homozygous for R462Q RNASEL Variant PLoS Pathog 2006 2 3 e25 10.1371/journal.ppat.0020025 16609730 PMC1434790

[B7] Wong C Heng C Wan Yee L Soh S Kartasasmita C Simoes E Hibberd M Sung W-K Miller L Optimization and clinical validation of a pathogen detection microarray Genome Biology 2007 8 5 R93 10.1186/gb-2007-8-5-r93 17531104 PMC1929155

[B8] Kessler N Ferraris O Palmer K Marsh W Steel A Use of the DNA Flow-Thru Chip, a Three-Dimensional Biochip, for Typing and Subtyping of Influenza Viruses J Clin Microbiol 2004 42 5 2173 2185 10.1128/JCM.42.5.2173-2185.2004 15131186 PMC404638

[B9] Victoria JG Wang C Jones MS Jaing C McLoughlin K Gardner S Delwart EL Viral nucleic acids in live-attenuated vaccines: detection of minority variants and an adventitious virus J Virol 2010 JVI.02690 02609 10.1128/JVI.02690-09 PMC2876658 20375174

[B10] DeSantis T Brodie E Moberg J Zubieta I Piceno Y Andersen G High-Density Universal 16S rRNA Microarray Analysis Reveals Broader Diversity than Typical Clone Library When Sampling the Environment Microbial Ecology 2007 53 3 371 383 10.1007/s00248-006-9134-9 17334858

[B11] Chou C-C Lee T-T Chen C-H Hsiao H-Y Lin Y-L Ho M-S Yang P-C Peck K Design of microarray probes for virus identification and detection of emerging viruses at the genus level BMC Bioinformatics 2006 7 1 232 10.1186/1471-2105-7-232 16643672 PMC1523220

[B12] Palacios G Quan P-L Jabado O Conlan S Hirschberg D Liu Y Panmicrobial oligonucleotide array for diagnosis of infectious diseases Emerg Infect Dis 2007 13 1 http://www.cdc.gov/NCIDOD/EID/13/1/73.htm 10.3201/eid1301.060837 17370518 PMC2725825

[B13] Sengupta S Onodera K Lai A Melcher U Molecular Detection and Identification of Influenza Viruses by Oligonucleotide Microarray Hybridization J Clin Microbiol 2003 41 10 4542 4550 10.1128/JCM.41.10.4542-4550.2003 14532180 PMC254299

[B14] Wang D Coscoy L Zylberberg M Avila PC Boushey HA Ganem D DeRisi JL Microarray-based detection and genotyping of viral pathogens Proceedings of the National Academy of Sciences of the United States of America 2002 99 24 15687 15692 10.1073/pnas.242579699 12429852 PMC137777

[B15] Wang X-W Zhang L Jin L-Q Jin M Shen Z-Q An S Chao F-H Li J-W Development and application of an oligonucleotide microarray for the detection of food-borne bacterial pathogens Applied Microbiology and Biotechnology 2007 76 1 225 233 10.1007/s00253-007-0993-x 17492283

[B16] Jin L-Q Li J-W Wang S-Q Chao F-H Wang X-W Yuan Z-Q Detection and identification of intestinal pathogenic bacteria by hybridization to oligonucleotide microarrays World J Gastroenterol 2005 11 48 7615 7619 16437687 10.3748/wjg.v11.i48.7615 PMC4727218

[B17] Anthony RM Brown TJ French GL Rapid Diagnosis of Bacteremia by Universal Amplification of 23S Ribosomal DNA Followed by Hybridization to an Oligonucleotide Array J Clin Microbiol 2000 38 2 781 788 10655385 10.1128/jcm.38.2.781-788.2000 PMC86203

[B18] Quan P-L Palacios G Jabado OJ Conlan S Hirschberg DL Pozo F Jack PJM Cisterna D Renwick N Hui J Detection of Respiratory Viruses and Subtype Identification of Influenza A Viruses by GreeneChipResp Oligonucleotide Microarray J Clin Microbiol 2007 45 8 2359 2364 10.1128/JCM.00737-07 17553978 PMC1951265

[B19] Jabado OJ Liu Y Conlan S Quan PL Hegyi H Lussier Y Briese T Palacios G Lipkin WI Comprehensive viral oligonucleotide probe design using conserved protein regions Nucl Acids Res 2008 36 1 e3 10.1093/nar/gkm1106 18079152 PMC2248741

[B20] Satya R Zavaljevski N Kumar K Reifman J A high-throughput pipeline for designing microarray-based pathogen diagnostic assays BMC Bioinformatics 2008 9 10.1186/1471-2105-9-185 PMC2375140 18402679

[B21] Takahashi K Iwasa Y Hijikata M Mishiro S Identification of a new human DNA virus (TTV-like mini virus, TMLM) intermediately related to TT virus and chicken anemia virus Archives of Virology 2000 145 979 993 10.1007/s007050050689 10881684

[B22] Li L Victoria JG Wang C Jones M Fellers GM Kunz TH Delwart E Bat Guano Virome: Predominance of Dietary Viruses from Insects and Plants plus Novel Mammalian Viruses J Virol 84 14 6955 6965 10.1128/JVI.00501-10 20463061 PMC2898246

[B23] Breitbart M Hewson I Felts B Mahaffy JM Nulton J Salamon P Rohwer F Metagenomic Analyses of an Uncultured Viral Community from Human Feces J Bacteriol 2003 185 20 6220 6223 10.1128/JB.185.20.6220-6223.2003 14526037 PMC225035

[B24] Urisman A Fischer K Chiu C Kistler A Beck S Wang D DeRisi J E-Predict: a computational strategy for species identification based on observed DNA microarray hybridization patterns Genome Biol 2005 6 9 R78 10.1186/gb-2005-6-9-r78 16168085 PMC1242213

[B25] Bailey T Gribskov M Combining evidence using p-values: application to sequence homology searches Bioinformatics 1998 14 1 48 54 10.1093/bioinformatics/14.1.48 9520501

[B26] Giegerich R Kurtz S Stoye J Efficient implementation of lazy suffix trees Software-Practice and Experience 2003 33 1035 1049 10.1002/spe.535

[B27] Rozen S Skaletsky HJ Krawetz S, Misener S Primer3 on the WWW for general users and for biologist programmers Bioinformatics Methods and Protocols: Methods in Molecular Biology 2000 Humana Press, Totowa, NJ 365 386 10.1385/1-59259-192-2:365 10547847

[B28] Markham NR Zuker M Keith JM UNAFold: software for nucleic acid folding and hybridization Bioinformatics, Volume II. Structure, Functions and Applications, number 453 in Methods in Molecular Biology 2008 II Totowa, NJ, Humana Press 3 31 10.1007/978-1-60327-429-6_1 18712296

[B29] Chiu CharlesÂ Y Rouskin S Koshy A Urisman A Fischer K Yagi S Schnurr D Eckburg PaulÂ B Tompkins LucyÂ S Blackburn BrianÂ G Microarray Detection of Human Parainfluenzavirus 4 Infection Associated with Respiratory Failure in an Immunocompetent Adult Clinical Infectious Diseases 2006 43 8 e71 e76 10.1086/507896 16983602 PMC7108001

[B30] Slezak T Kuczmarski T Ott L Torres C Medeiros D Smith J Truitt B Mulakken N Lam M Vitalis E Comparative genomics tools applied to bioterrorism defense Briefings in Bioinformatics 2003 4 2 133 149 10.1093/bib/4.2.133 12846395

