# Supplementary material for: A microbial detection array (MDA) for viral and bacterial detection
Source: BMC Genomics. 2010 Nov 25;11:668. doi: 10.1186/1471-2164-11-668 (PMC3017867; doi:10.1186/1471-2164-11-668)
Supplement: Additional file 1 — Primer sequences and product sizes used to confirm the array results from clinical samples. [file 1471-2164-11-668-S1.DOCX]

Table S1: Primer sequences and product sizes used to confirm the array results from clinical samples.

| **Sample** | **Forward Primer** | **Reverse Primer** | **Expected Product Size (EPS)** | **EPS Detected** |
| --- | --- | --- | --- | --- |
| **DeRset1_1** |  |  |  |  |
| Coronavirus HKU1 | CTATGAAGTCAGATGAGGGTGGG | GAACGGAACAAGCCCATAACATA | 287 | Yes |
| RSV | GGCAAATATGGAAACATACGTGAA | GACTCGTAGTGAAGGTCCTTTGG | 224 | Yes |
|  |  |  |  |  |
| **DeRset2_1** |  |  |  |  |
| Human parechovirus 1 isolate BNI-788St | AGATACCACGCTTGTGGACCTTA | GGGTTTGTTAAACCTTGGCTTTT | 180 | Yes |
| Streptococcus thermophilus LMD9 | CGTATCTGCCCGTATGCTTG | CGCCCCAAACAAAGAATAGC | 265 | Yes |
|  |  |  |  |  |
| **DeRset2_2** |  |  |  |  |
| Escherichia coli CFT073 | ATCCGTCATACGGAACATCAACT | AGAGAAAACGGAAGAGTATCGCC | 144 | Yes |
| Norwalk virus 1 | GCTCCCAGTTTTGTGAATGAAGA | CACCATCATTAGATGGAGCGG | 60 | Yes |
| Norwalk virus 2 | TTCACAAAACTGGGAGCC | ATGGACTTTTACGTGCC | 105 | Yes |
|  |  |  |  |  |
| **DeRset2_3** |  |  |  |  |
| Chicken anemia virus | GTTCAGGCCACCAACAAGTTC | TTAGCTCGCTTACCCTGTACTCG | 258 | Yes |
| Serratia proteamaculans 1 | CCGCAGATCCTGGCTAAAA | GCCGAATCAACGAAGCCTAC | 203 | No |
| Serratia proteamaculans 2 | CCCTGGGTAAGGTGAAAACG | CCCATAGCACCGCTTATCCT | 221 | No |
|  |  |  |  |  |
| **DeRset2_4** |  |  |  |  |
| Staphylococcus aureus | CATGCGTATTGCTATTGAGTTGC | ATGCAAACGAGTCCAAGCAG | 281 | Yes |
| Shigella & E.coli conserved region | CGTCTGCTGGATGGCTTCTA | TCTCTTCTTCCGGCACCATT | 239 | Yes |
| Shigella sonnei Ss046 plasmid pSS046_spB | GGGTGGAAAAGTTGGGATCA | GGCTCTGGAGCAGGAAAAGA | 287 | Yes |
| Lactococcus lactis pGdh442 plasmid | AGGTGACCGTACTTTACACAATGG | TTCGCTTGTGTTCGTCCTTG | 276 | Yes |
| Streptococcus sanguinis | AACGAGCTGTTGAGGGCAAT | TATGTACGGCGTCAAGGAGC | 300 | Yes |
| Lactococcus lactis pCI305 plasmid | TGGAAAATTGCGTCCTTATTTG | TCGAGGGAACTGGGAATTTG | 232 | Yes |
| E.coli pAPEC O2-ColV plasmid 1 | CGGACGGCTACTGAACCAAT | ATGCCTGCTCAACTCCATCA | 255 | No |
| E.coli pAPEC O2-ColV plasmid 2 | GCAGAAATGAAGCTGATGCG | CTGAAGGCCATCACCCGT | 82 | No |
